# Effect of maternal body mass index on the steroid profile in women with gestational diabetes mellitus

**DOI:** 10.3389/fendo.2022.999154

**Published:** 2022-11-09

**Authors:** Yanni Sun, Bo Zhu, Xingjun Meng, Binbin Yin, Kaiqi Wu, Yifeng Liu, Dandan Zou, Jianyou Xue, Xiao Sun, Dan Zhang, Zhixin Ma

**Affiliations:** ^1^ Women’s Hospital, School of Medicine, Zhejiang University, Hangzhou, China; ^2^ Clinical Prenatal Diagnosis Center, Women’s Hospital, School of Medicine, Zhejiang University, Hangzhou, China; ^3^ Key Laboratory of Women’s Reproductive Health of Zhejiang Province, and Women’s Hospital, School of Medicine, Zhejiang University, Hangzhou, China; ^4^ Hangzhou BIOZON Medical Laboratory co. Ltd., Hangzhou, Zhejiang, China

**Keywords:** gestational diabetes mellitus, steroid hormone, body mass index, estrogen, androgen

## Abstract

**Objective:**

To explore the effect of maternal body mass index (BMI) on steroid hormone profiles in women with gestational diabetes mellitus (GDM) and those with normal glucose tolerance (NGT).

**Methods:**

We enrolled 79 women with NGT and 80 women with GDM who had a gestational age of 24–28 weeks. The participants were grouped according to their BMI. We quantified 11 steroid hormones profiles by liquid chromatography-tandem mass spectrometry and calculated the product-to-precursor ratios in the steroidogenic pathway.

**Results:**

Women with GDM and BMI<25kg/m^2^ showed higher concentrations of dehydroepiandrosterone (DHEA) (*p*<0.001), testosterone (T) (*p=*0.020), estrone (E1) (*p*=0.010) and estradiol (E2) (*p*=0.040) and lower Matsuda index and HOMA-β than women with NGT and BMI<25kg/m^2^. In women with GDM, concentrations of E1 (*p*=0.006) and E2 (*p*=0.009) declined, accompanied by reduced E2/T (*p*=0.008) and E1/androstenedione (A4) (*p*=0.010) in the BMI>25 kg/m^2^ group, when compared to that in the BMI<25 kg/m^2^ group. The values of E2/T and E1/A4 were used to evaluate the cytochrome P450 aromatase enzyme activity in the steroidogenic pathway. Both aromatase activities negatively correlated with the maternal BMI and positively correlated with the Matsuda index in women with GDM.

**Conclusions:**

NGT women and GDM women with normal weight presented with different steroid hormone profiles. Steroidogenic pathway profiling of sex hormones synthesis showed a significant increase in the production of DHEA, T, E1, and E2 in GDM women with normal weight. Additionally, the alteration of steroid hormone metabolism was related to maternal BMI in women with GDM, and GDM women with overweight showed reduced estrogen production and decreased insulin sensitivity compared with GDM women with normal weight.

## 1 Introduction

Gestational diabetes mellitus (GDM) is defined as glucose intolerance and hyperglycemia that occur during pregnancy. It is one of the most common complications during pregnancy, which seriously threatens maternal and fetal health (1, 2). According to clinical statistics, at least 30% of women with a history of GDM are likely to develop type 2 diabetes mellitus (T2DM) after delivery (3). The risk of T2DM in pregnant women with GDM is approximately seven times higher than that in pregnant women without GDM (4). The incidence of macrosomia in the offspring of pregnant women with GDM is approximately 15-45%, which is three times higher than that in healthy pregnant women (5). It has been reported that increasing maternal body mass index (BMI) is an independent risk factor for the development of GDM (6). During pregnancy, excessive weight gain and higher maternal BMI may result in increased insulin resistance and further exacerbate maternal hyperglycemia (7). Greater fat deposition may reduce the ability to compensate for the physiological increase in insulin resistance that occurs during gestation (8, 9). A number of interrelated factors including overweight/obesity and steroids affecting both insulin secretion and insulin resistance are involved in the pathophysiology of GDM (10).

Abnormal metabolism of steroid hormones may induce physiological disorders that lead to complications in obstetrics and gynecology, such as infertility, miscarriage, polycystic ovary syndrome (PCOS), preeclampsia, and GDM (11–13). It has been reported that the pancreas is a target of gonadal steroids, and steroids metabolites have been shown to regulate pancreatic function and insulin resistance in T2DM (14, 15). Progression of pregnancy is accompanied by significant changes in steroid hormones (16). At the beginning of pregnancy, ovarian corpus luteum cells play an essential role in progesterone production. As the placenta develops during pregnancy, the levels of various maternal hormones, including lactogen, placental prolactin, glucocorticoids, estrogen, and androgen, begin to rise rapidly at 24-28 weeks of gestation, while insulin sensitivity starts to decline simultaneously, which promote a state of insulin resistance (17, 18).

Hyperglycemia during pregnancy is the result of impaired glucose tolerance caused by pancreatic β-cell dysfunction on a background of chronic insulin resistance (10). Previous studies have shown that serum dehydroepiandrosterone sulfate (DHEAS) levels may directly affect beta cell function by enhancing glucose-stimulated insulin secretion and specific mRNA expression of beta cell mitochondria and peroxisomal lipid metabolic enzymes (19). Dokras et al. found that testosterone (T) levels in pregnant women were positively correlated with insulin responses during a glucose tolerance test (20). In addition, low levels of sex hormone-binding globulin (SHBG) in the first trimester are associated with an increased risk of developing GDM diagnosed in the second trimester (21). A clinical study has shown that T2DM and GDM are associated with specific changes in sexual steroids and insulin resistance levels during pregnancy. Hyperandrogenemia and higher insulin resistance is observed in women with pregestational T2DM, but not in women with GDM during pregnancy. Decreased estrogen and aromatase activity were found in women with pregestational T2DM and GDM during gestation (22). These studies showed that steroid hormones are related with insulin resistance and GDM development. Another study demonstrated a different metabolic profile of steroid hormones in lean and obese PCOS patients; in that, excessive androgen accumulation was observed in obese PCOS patients with higher insulin resistance than in lean ones (23). Additionally, maternal BMI is a known risk factor for GDM. However, whether the change in maternal weight or BMI has any effect on the steroid hormone profiles in women with GDM and normal pregnant women has not been reported.

The aim of the present study was to explore the difference in steroid profiles in GDM patients and normal pregnant women at 24–28 gestational weeks and to investigate the effect of maternal BMI on steroid hormone profiles and steroid metabolic pathway in women with GDM.

## 2 Materials and methods

### 2.1 Study population and sample collection

**Figure 1:**
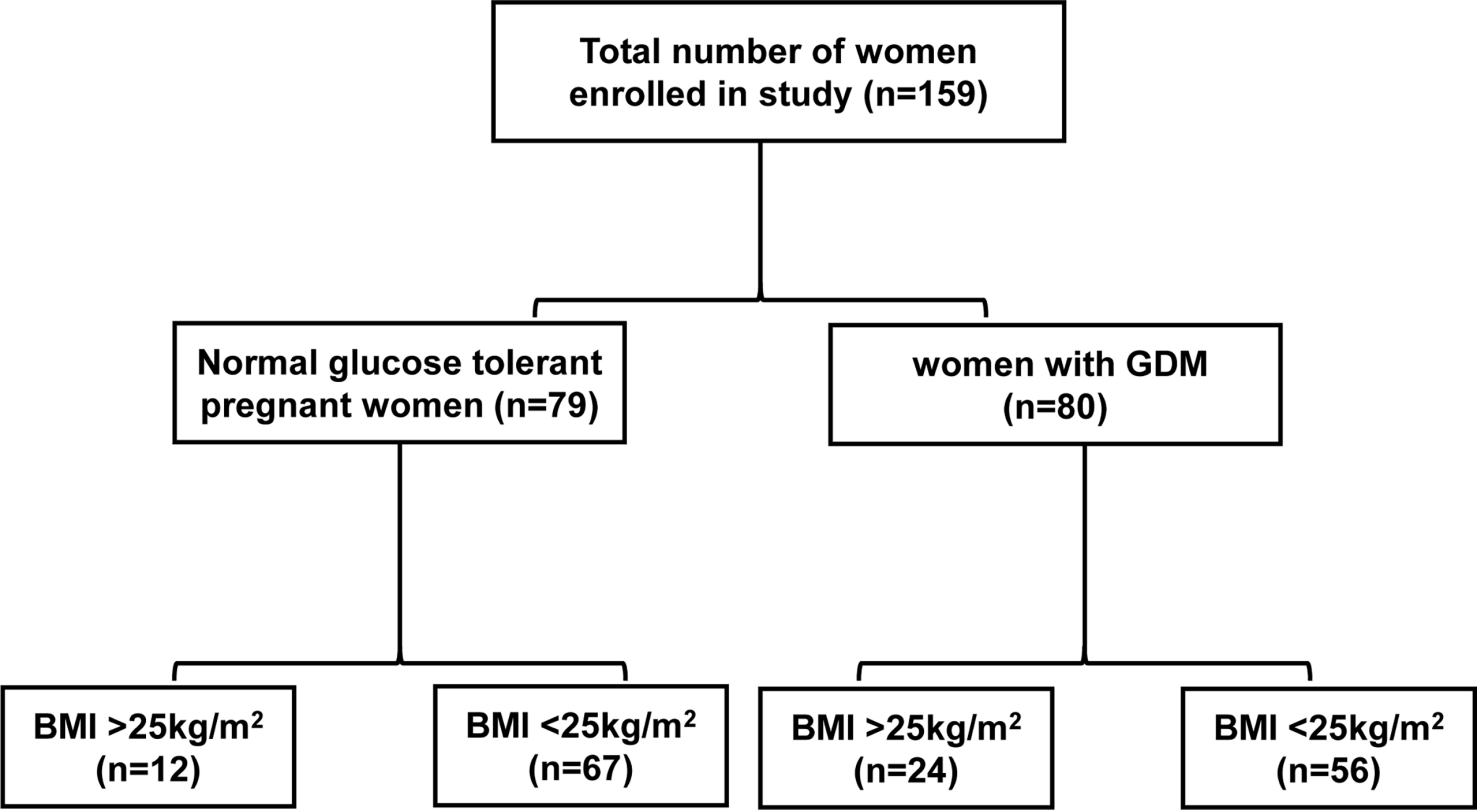
Flow chart of the study population.

Eighty GDM patients and 79 pregnant women with normal glucose tolerance (NGT) with a gestational age of 24–28 weeks were enrolled between April 7, 2020, and May 22, 2020, at the Women’s Hospital of Zhejiang University School of Medicine, China. The pregnant women were between 23 and 35 years old, with a single fetus and normal fetal development at gestational age of 24–28 weeks. The exclusion criteria were as follows (1): *in vitro* fertilization embryo transfer (IVF-ET) (2); personal history of chronic diseases, type 1 Diabetes Mellitus and T2DM, PCOS, autoimmune or chromosomal diseases and liver, kidney, adrenal or thyroid dysfunction (3); diseases that require hormone therapy. First, we compared the differences between women with GDM and those with NGT. The participants were subdivided according to BMI: BMI>25kg/m^2^ GDM group (n=24), BMI<25kg/m^2^ GDM group (n=56), BMI>25kg/m^2^ NGT group (n=12), and BMI<25kg/m^2^ NGT group (n=67) (**Figure 1**). Fasting venous blood sample were collected after 8–14 hours of fasting at the date with oral glucose tolerance test (OGTT). Blood samples were left at room temperature for 30 min, and the upper serum was separated after centrifugation. Finally, serum was stored at -80°C for the detection of steroid hormones. This study was approved by the Ethics Committee of Women’s Hospital School of Medicine, Zhejiang University (IRB-20200305-R).

### 2.2 GDM diagnostic criteria

We used 75-g OGTT at 24–28 gestational weeks for the diagnostic criteria of GDM, which is recommended by the International Association of Diabetes and Pregnancy Study Group (IADPSG) (24). A pregnant woman who meets one of the following conditions may be diagnosed with GDM (1): fasting glucose ≥5.1 mmol/L (2); 1 h glucose ≥10.0 mmol/L (3); 2 h glucose ≥8.5 mmol/L.

### 2.3 Methods of steroid hormones detection

#### 2.3.1 Sample preparation

In our experiment, calibrators (Bepure, CHN) were dissolved in 10% methanol and 90% water. Internal standard (Bepure, CHN) was dissolved in methanol. Firstly, 200 μL methanol was added to an HLB SPE plate (Waters, Oasis PRiME HLB 96-well µElution Plate, USA), and was lowly flowed through the plate under low vacuum. Thereafter, 200 μL water was flowed through the SPE plate to balance the plate, after which the 200 μL calibrators, quality controls (QCs), and serum samples were placed into a 1.5 mL tube, wherein 200 μL of an internal standard mixture was added and mixed for 3 min. Finally, we added 400μL water, mixed it for 1min, and centrifuged it at 4°C for 15000 g for 10 min. The supernatant (700 μL) was then added to the SPE plates. A low vacuum causes the supernatant to flow slowly through the SPE plate. The HLB SPE plate was washed once with 200 μL 15% methanol. After 60μL of methanol was eluted into a 96-well plate and mixed with 60μL of water, the extract was analyzed by liquid chromatography-tandem mass spectrometry (LC-MS/MS). The details on the methods are shown in **Supplementary Materials Tables 1–5**. The recovery experiment and matrix effect are referred to literatures (25, 26).

#### 2.3.2 LC-MS/MS

LC column: Waters HSS T3 (2.1×50 mm, 1.8 μm), pre column: HSS T3 (2.1×5.0 mm, 1.8 μm).

LC method A: The mobile phase consisted of 2 mM ammonium acetate, 0.1% formic acid, and water (solvent A) or methanol (solvent B). The liquid chromatographic gradient was as follows: at 0~4 min, 40%~60% solvent B; 4~6.5 min, 60%~75% solvent B; 6.5~7.5 min, 75%~90% solvent B; 7.5~7.6 min, 90%~45% solvent B; and 7.6~8.0 min, 40% solvent B. Column temperature was 45°C, injection volume was 10 μL and flow rate was 0.45 mL/min.

LC method B: The mobile phase consisted of a 0.1% ammonia solution and water (solvent A) or methanol (solvent B). The liquid chromatographic gradient was as follows: at 0~0.6 min, 30% solvent B; 0.6~0.7 min, 30%~65% solvent B; 0.7~1.5 min, 65~85% solvent B; 1.5~2.5 min, 85%~98% solvent B; 2.5~2.6 min, 98%~30% solvent B; and 2.6~3.0 min, 30% solvent B. Column temperature was 45°C, injection volume was 10 μL and flow rate was 0.4 mL/min.

Detection was performed using LC-MS/MS (Waters TQS) equipped with an electrospray ionization probe and operated by switching between positive and negative ionization modes. The capillary potential was set at 3.2 kV. The ion-source temperature was 150°C and the desolvation gas was heated to 400°C at a flow rate of 600 L/h. The cone gas flow rate was set to 150 L/h. Multiple reaction monitoring was used for quantification, as listed in **Supplementary Materials Table 6**. Data acquisition was achieved using Masslynx software.

### 2.4 Statistical analysis

SPSS software version 22 (SPSS Inc., Chicago, IL, USA) was used for the data analysis. The student’s t-test was performed using the clinical characteristics between the two groups. The data are presented as mean ± standard error (SEM). The comparison of steroid hormone profiling and product/precursor ratios was made using the non-parametric test of Mann–Whitney U. The data are presented as median (25,75 percentile). *p*<0.05 was considered statistically significant. Spearman correlation analysis was also performed. * *p*<0.05, ** *p*<0.01.

Matsuda index=10000/(G_0_×I_0_) ^½^ (G_mean_×I_mean_) ^½^ and HOMA-β=20× I_0_/(G_0_-3.5), where G_0_ is fasting glucose (mmol/L) and I_0_ is fasting insulin (μU/mL). G_mean_ (mmol/L) is the mean of fasting glucose, 1-h glucose OGTT, and 2-h glucose OGTT. I_mean_ (μU/mL) is the mean of fasting insulin, 1-h insulin OGTT, and 2-h insulin OGTT. Matsuda index and HOMA-β data were log transformed to meet normality.

## 3 Results

### 3.1 Baseline characteristics and steroid profiles of women with GDM and women with NGT

Women with GDM (n=80) and women with NGT (n=79) had similar maternal age, gestational age, blood pressure, total cholesterol, HDL-cholesterol and LDL-cholesterol. Women with GDM had a significantly higher level of fasting glucose (*p*<0.001), 1-h glucose (*p*<0.001), 2-h glucose (*p*<0.001), 2-h insulin (*p*<0.001), hemoglobin A1c (HbA1c) (*p*=0.040) and triacylglycerol (TG) (*p*=0.03) than women with NGT. The indexes related with insulin resistance including Matsuda index (*p*<0.001) and Homeostasis model assessment (HOMA)-β (*p*=0.045) were decreased in women with GDM (shown in **Table 1**). Additionally, using LC-MS/MS, we compared the serum levels of 11 steroid hormones between the two groups, including pregnenolone(P5), progesterone (P4), 17α-hydroxypregnenolone (17OHP5), 17α-hydroxyprogesterone (17OHP4), dehydroepiandrosterone (DHEA), androstenedione (A4), T, dihydrotestosterone (DHT), estriol (E3), estradiol (E2), and estrone (E1). Compared with women with NGT, women with GDM showed significantly higher concentrations of DHEA (*p*=0.001), A4 (*p*=0.023), and T (*p*<0.001) (shown in **Table 2**). Further analysis indicated an elevated risk of GDM in women with high levels (greater than median) of DHEA (OR=2.582, *p*=0.003) and T (OR=2.725, *p*=0.002) compared with those with low levels (less than or equal to median) (**Table 3**). Surprisingly, we found no significant differences in maternal BMI between the two groups. For a better understanding of the alterations in steroid hormone metabolism in women with GDM and NGT, we subdivided the participants by BMI.

**Table 1:** Baseline characteristics in women with GDM and women with NGT.

	NGT group (n=79)	GDM group (n=80)	*p* Value
**Clinical measures**			
Maternal age (year)	28.94 ± 0.32	30.26 ± 0.30	ns
Maternal BMI (kg/m^2^)	23.16 ± 0.23	23.49 ± 0.31	ns
Gestational age (weeks)	24.87 ± 0.13	25.15 ± 0.13	ns
Systolic blood pressure (mm Hg)	112.35 ± 1.85	113.72 ± 1.21	ns
Diastolic blood pressure (mm Hg)	65.82 ± 1.14	66.35 ± 0.95	ns
Fasting glucose (mmol/L)	4.38 ± 0.03	4.74 ± 0.06	<0.001
1-h glucose OGTT (mmol/L)	7.54 ± 0.11	10.85 ± 0.15	<0.001
2-h glucose OGTT (mmol/L)	6.48 ± 0.85	9.59 ± 0.14	<0.001
Fasting insulin (μU/mL))	7.60 ± 0.81	8.61 ± 0.47	ns
1-h insulin OGTT (μU/mL))	57.34 ± 3.14	59.24 ± 3.29	ns
2-h insulin OGTT (μU/mL))	45.79 ± 2.35	71.17 ± 4.18	<0.001
HbA1c (%)	4.94 ± 0.03	5.12 ± 0.05	0.040
TG (mmol/l)	2.03 ± 0.08	2.51 ± 0.11	0.030
Total cholesterol (mmol/L)	5.89 ± 0.10	6.12 ± 0.12	ns
HDL-cholesterol (mmol/L)	1.87 ± 0.04	1.82 ± 0.04	ns
LDL-cholesterol (mmol/L)	3.26 ± 0.08	3.34 ± 0.10	ns
Matsuda index	138.02 ± 5.78	97.79 ± 5.25	<0.001
HOMA-β	180.36 ± 16.94	133.55 ± 25.40	0.045

The student’s t-test was performed between the two groups. All data are presented as mean ± standard error (SEM), p<0.05 was considered statistically significant. ns means that there is no significant difference between the two groups. Matsuda index and HOMA-β data were log transformed to meet normality. Matsuda index=10000/(G_0_×I_0_)^1/2^ (G_mean_×I_mean_) ^1/2^.HOMA-β=20× I_0_/ (G_0_-3.5). G_0_ is fasting glucose (mmol/L), I_0_ is fasting insulin (μU/mL). G_mean_ (mmol/L) is the mean of fasting glucose, 1-h glucose OGTT and 2-h glucose OGTT. I_mean_(μU/mL) is the mean of fasting insulin, 1-h insulin OGTT and 2-h insulin OGTT.

**Table 2:** The results of steroid hormones in women with GDM and women with NGT.

	NGT group (n=79)	GDM group (n=80)	*p* Value
Steroid hormones profiling (ng/mL)			
Pregnenolone (P5)	1.11 (0.86,1.42)	1.14 (0.94,1.48)	ns
Progesterone (P4)	48.61 (40.89,59.18)	52.92 (45.13,60.76)	ns
17α-Hydroxypregnenolone (17OHP5)	1.09 (0.88,1.42)	1.17 (0.87,1.53)	ns
17α-Hydroxyprogesterone (17OHP4)	2.81 (2.27,3.48)	3.12 (2.53,3.62)	ns
Dehydroepiandrosterone (DHEA)	1.84 (1.50,2.19)	2.39 (1.77,3.29)	0.001
Androstenedione (A4)	3.38 (2.57,4.29)	3.57 (2.75,6.01)	0.023
Testosterone (T)	0.69 (0.54,0.89)	0.87 (0.65,1.52)	<0.001
Dihydrotestosterone (DHT)	0.014 (0.011,0.018)	0.015 (0.01,0.02)	ns
Estrone (E1)	0.07 (0.05,0.10)	0.08 (0.06,0.11)	ns
Estradiol (E2)	0.39 (0.32,0.45)	0.39 (0.32,0.52)	ns
Estriol (E3)	0.09 (0.08,0.11)	0.10 (0.08,0.11)	ns

Mann-Whitney U test was performed between the two groups. All data are presented as median (25,75 percentile). p<0.05 was considered statistically significant. ns means that there is no significant difference between the two groups.

**Table 3:** Calculated odds ratio (OR) for GDM.

Steroid hormones	Odds ratio	95% confidence limits	*p* Value
DHEA (high versus low)	2.582	(1.362, 4.893)	0.003
A4 (high versus low)	1.388	(0.744, 2.590)	0.302
T (high versus low)	2.725	(1.435, 5.176)	0.002

“High” is the level of steroid greater than median; ‘low’ is the level of steroid less than or equal to median). OR and p value were obtained by Chi-square test. p<0.05 was considered statistically significant.

### 3.2 Analysis of clinical characteristics and steroid profiles of GDM women and NGT women with normal weight

We observed that the GDM group (BMI<25 kg/m^2^) had higher maternal age (*p*=0.040) and higher serum levels of fasting glucose (p<0.001), 1-h glucose (*p*<0.001), 2-h glucose (*p*<0.001), 2-h insulin (*p*<0.001), and TG (*p*=0.007) when compared to the NGT group (BMI<25 kg/m^2^). Matsuda index and HOMA-β were decreased in the GDM group (**Table 4**). The levels of DHEA (*p*<0.001), T (*p*=0.020), E1 (*p*=0.010), and E2 (*p*=0.040) were increased in the GDM group (BMI<25 kg/m^2^) compared to those in the NGT group (BMI<25 kg/m^2^). Next, we used the ratio of product-to-precursor in the steroid hormone metabolic pathway to demonstrate the activity of the enzymes involved in the reaction. The results indicated that DHEA/17OHP5 (*p*=0.010) and T/A4 (*p*=0.003) were increased and DHT/T was decreased in the GDM group (*p*<0.001) (**Table 5**).

**Table 4:** Clinical characteristics of GDM group and NGT groups with different maternal BMI.

	NGT		GDM			
	BMI<25 (n=67)^a^	BMI>25(n=12)	BMI<25 (n=56)^b^	BMI>25(n=24)^c^	P VALUE (a VS. b)	P VALUE (b VS. c)
**Clinical measures**						
Maternal age (year)	29.06 ± 0.36	28.25 ± 0.54	30.07 ± 0.33	30.71 ± 0.63	0.04	ns
Maternal BMI (kg/m^2^)	22.60 ± 0.21	26.25 ± 0.26	22.02 ± 0.24	26.91 ± 0.27	ns	<0.001
Gestational age (weeks)	24.71 ± 0.12	25.78 ± 0.41	24.98 ± 0.15	25.53 ± 0.24	ns	ns
Systolic blood pressure (mm Hg)	112.70 ± 1.59	110.17 ± 8.62	111.90 ± 1.41	117.80 ± 2.07	ns	ns
Diastolic blood pressure (mm Hg)	65.30 ± 1.27	68.75 ± 2.42	64.52 ± 1.12	70.25 ± 1.52	ns	0.023
Fasting glucose(mmol/L)	4.35 ± 0.03	4.53 ± 0.09	4.61 ± 0.06	5.03 ± 0.12	<0.001	0.001
1-h glucose OGTT (mmol/L)	7.50 ± 0.12	7.75 ± 0.26	10.78 ± 0.18	11.02 ± 0.31	<0.001	ns
2-h glucose OGTT (mmol/L)	6.47 ± 0.08	6.52 ± 0.30	9.67 ± 0.15	9.41 ± 0.32	<0.001	ns
Fasting insulin (μU/mL))	7.19 ± 0.94	9.81 ± 1.02	7.27 ± 0.36	11.74 ± 1.12	ns	<0.001
1-h insulin OGTT (μU/mL)	56.03 ± 3.29	64.7 ± 9.67	52.50 ± 3.09	74.96 ± 7.42	ns	0.001
2-h insulin OGTT (μU/mL)	44.64 ± 2.45	52.24 ± 7.28	63.20 ± 3.92	89.79 ± 9.64	<0.001	0.003
HbA1C (%)	4.92 ± 0.03	5.04 ± 0.06	5.01 ± 0.05	5.36 ± 0.07	ns	<0.001
Triacylglycerides (mmol/L)	1.96 ± 0.08	2.39 ± 0.32	2.39 ± 0.14	2.78 ± 0.16	0.007	ns
Total cholesterol (mmol/L)	5.87 ± 0.10	6.00 ± 0.39	6.20 ± 0.12	5.90 ± 0.28	ns	ns
HDL-cholesterol (mmol/L)	1.85 ± 0.04	1.99 ± 0.14	1.86 ± 0.05	1.71 ± 0.06	ns	ns
LDL-cholesterol (mmol/L)	3.27 ± 0.09	3.20 ± 0.29	3.41 ± 0.11	3.19 ± 0.22	ns	ns
Matsuda index	143.65 ± 6.25	106.61 ± 11.99	106.27 ± 5.20	78.01 ± 12.19	<0.001	<0.001
HOMA-β	173.90 ± 18.87	216.70 ± 36.37	120.50 ± 35.59	163.90 ± 16.18	0.045	ns

p value (a VS. b) is the p value of women with NGT (BMI<25kg/m^2^) compared with women with GDM (BMI<25kg/m^2^). p value (b VS. c) is the p value of women with GDM (BMI<25kg/m^2^) compared with women with GDM (BMI>25kg/m^2^). The student’s t-test was performed. All data are presented as mean ± standard error (SEM), p<0.05 was considered statistically significant. ns means that there is no significant difference between the two groups.

**Table 5:** Steroid hormones profiling and product/precursor ratios of GDM group and NGT group with different maternal BMI.

	NGT		GDM					
	BMI<25(n=67)^a^	BMI>25(n=12)	BMI<25s(n=56)^b^		BMI>25(n=24)^c^	P VALUE (a VS. b)		P VALUE (b VS. c)
**Steroid hormones profiling (ng/mL)**								
Pregnenolone (P5)	1.13 (0.88,1.42)	1.09 (0.74,1.37)	1.22 (0.98,1.62)		0.97 (0.75,1.20)	0.03		0.001
Progesterone (P4)	48.61 (40.74,58.95)	48.63 (41.89,64.94)	54.41 (48.50,62.61)		46.76 (42.48,55.13)	0.02		0.009
17α-Hydroxypregnenolone (17OHP5)	1.09 (0.88,1.33)	1.19 (0.85,1.50)	1.22 (0.93,1.56)		1.02 (0.72,1.43)	ns		ns
17α-Hydroxyprogesterone (17OHP4)	2.79 (2.22,3.47)	2.93 (2.61,4.17)	3.13 (2.55,3.74)		3.06 (2.53,3.47)	ns		ns
Dehydroepiandrosterone (DHEA)	1.75 (1.47,2.15)	2.05 (1.78,3.12)	2.49 (1.78,3.41)		2.06 (1.61,3.24)	<0.001		ns
Androstenedione (A4)	3.38 (2.57,4.27)	3.41 (2.52,4.83)	3.57 (2.75,5.59)		3.87 (2.73,8.43)	ns		ns
Testosterone (T)	0.69 (0.53,0.89)	0.70 (0.55,0.88)	0.84 (0.63,1.43)		0.98 (0.72,1.72)	0.020		ns
Dihydrotestosterone (DHT)	0.014 (0.011,0.019)	0.01 (0.013,0.015)	0.015 (0.01,0.02)		0.014 (0.01,0.02)	ns		ns
Estrone (E1)	0.07 (0.05,0.10)	0.07 (0.06,0.11)	0.08 (0.06,0.12)		0.06 (0.05,0.08)	0.010		0.006
Estradiol (E2)	0.39 (0.31,0.46)	0.36 (0.33,0.43)	0.40 (0.35,0.59)		0.35 (0.27,0.42)	0.040		0.009
Estriol (E3)	0.09 (0.07,0.11)	0.09 (0.08,0.10)	0.10 (0.08,0.12)		0.10 (0.08,0.11)	ns		ns
**Product/precursor ratio**								
P4/P5	43.70 (35.63,63.23)	56.98 (37.58,65.52)		43.27 (32.78,56.61)	51.67 (37.13,64.68)		ns	ns
17OHP4/17OHP5	2.48 (1.95,3.05)	2.81 (1.49,3.80)		2.48 (1.95,3.53)	3.02 (2.03,4.69)		ns	ns
17OHP4/P4	0.05 (0.04,0.07)	0.06 (0.05,0.07)		0.06 (0.05,0.07)	0.07 (0.06,0.08)		ns	ns
A4/17OHP4	1.12 (0.90,1.44)	1.17 (0.98,1.22)		1.25 (0.98,1.60)	1.41 (1.11,2.28)		ns	ns
17OHP5/P5	1.01 (0.66,1.40)	1.18 (0.83,1.39)		0.94 (0.74,1.18)	1.18 (0.72,1.43)		ns	ns
DHEA/17OHP5	1.79 (1.27,2.15)	1.83 (1.28,2.72)		2.04 (1.70,2.47)	2.30 (1.73,2.72)		0.010	ns
A4/DHEA	1.62 (1.21,2.44)	1.36 (1.10,2.46)		1.44 (1.09,2.39)	2.18 (1.32,3.34)		ns	ns
T/A4	0.21 (0.19,0.23)	0.20 (0.18,0.23)		0.24 (0.20,0.28)	0.23 (0.21,0.27)		0.003	ns
DHT/T	0.020 (0.017,0.029)	0.018 (0.016,0.019)		0.016 (0.013,0.020)	0.013 (0.011,0.017)		<0.001	ns
E1/A4	0.022 (0.014,0.029)	0.024 (0.016,0.027)		0.022 (0.015,0.033)	0.013 (0.001,0.029)		ns	0.010
E2/E1	5.46 (4.46,6.69)	5.55 (3.44,6.06)		4.99 (3.96,6.14)	5.17 (4.55,6.70)		ns	ns
E3/E2	0.24 (0.20,0.29)	0.26 (0.19,0.30)		0.23 (0.18,0.30)	0.27 (0.24,0.37)		ns	0.003
E2/T	0.55 (0.41,0.76)	0.59 (0.42,0.71)		0.50 (0.31,0.71)	0.33 (0.24,0.49)		ns	0.008

Mann-Whitney U test was performed. All data are presented as median (25,75 percentile). p<0.05 was considered statistically significant. ns means that there is no significant difference between the two groups.

### 3.3 Analysis of clinical characteristics and steroid profiles of GDM women with normal weight and overweight

In the GDM group, women with BMI>25 kg/m^2^ had significantly higher levels of diastolic blood pressure (*p*=0.023), fasting glucose (*p*=0.001), fasting insulin (*p*<0.001), 1-h insulin (*p*=0.001), 2-h insulin (*p*=0.003) and HbA1c (*p*<0.001) than those with BMI<25 kg/m^2^ (**Table 4**). The Matsuda index was lower in the BMI>25 kg/m^2^ group than in the BMI<25 kg/m^2^ group in women with GDM. The between-group differences in HOMA-β did not reach significance.

Steroid hormone analysis showed that E1 (*p*=0.006) and E2(*p*=0.009) levels were significantly decreased in GDM women with BMI>25 kg/m^2^. Regarding enzymatic activity, the BMI>25 kg/m^2^ group showed an increased ratio of E3/E2 (*p*=0.003) and a decreased ratio of E1/A4 (p=0.010) and E2/T (p=0.008) compared to the BMI<25 kg/m^2^ group (**Table 5**). No difference was observed in NGT women with normal weight and overweight (**Supplementary Materials Figure 1**).

### 3.4 Association between steroid hormones ratio with BMI in women with GDM

**Figure 2:**
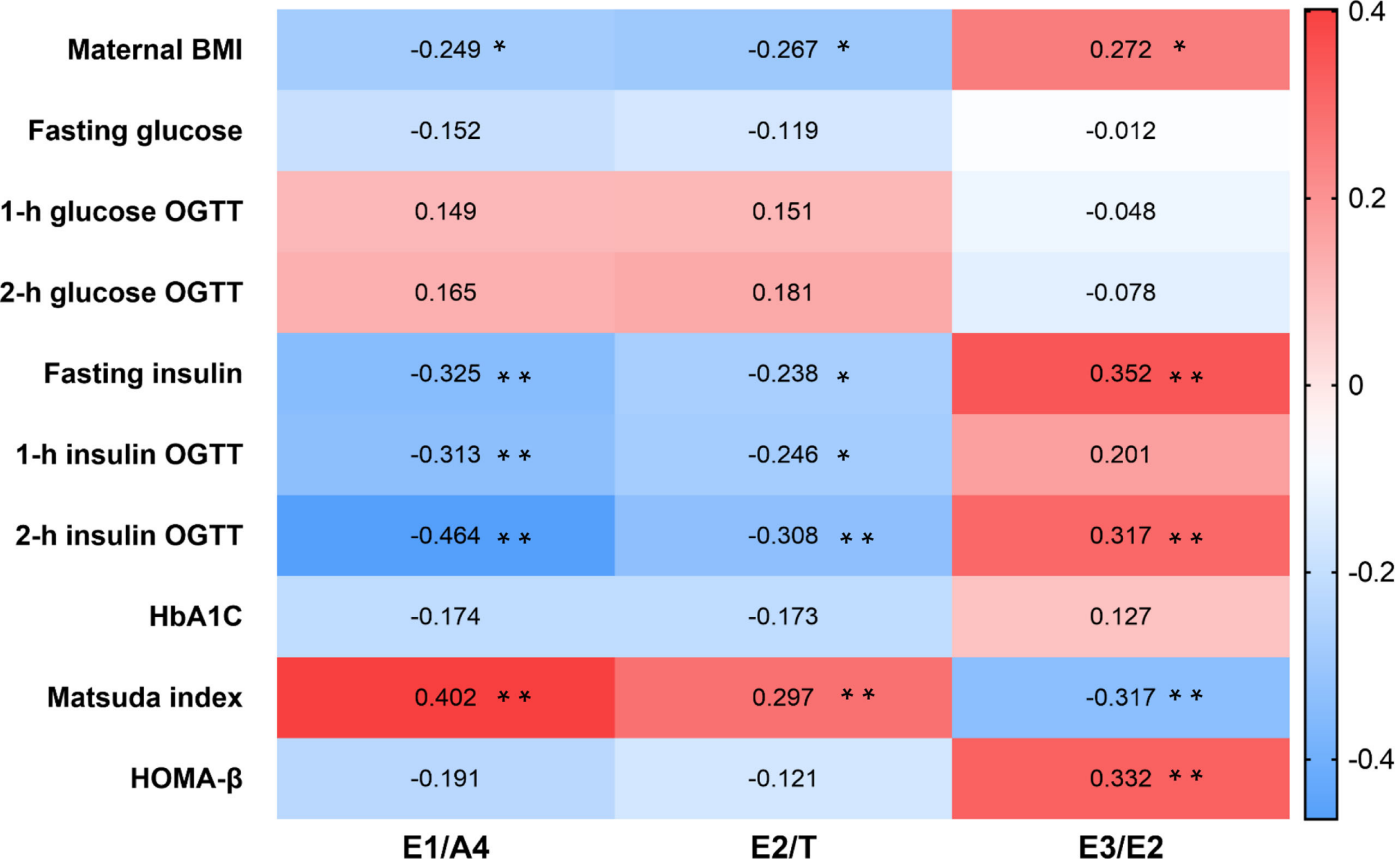
Heatmap of steroid hormones ratio and clinical characteristics in GDM patients. Correlation between steroid hormone ratio and clinical data in women with GDM using Spearman correlation analysis. *p < 0.05, **p < 0.01. GDM, gestational diabetes mellitus.

Correlation analysis in the GDM cohort indicated that BMI was negatively correlated with E1/A4 (r=-0.249, *p*=0.026) and E2/T (r=-0.267, *p*=0.016). The Matsuda index was positively correlated with E1/A4 (r=0.402, *p*<0.001) and E2/T (r=0.297, *p*=0.007). In addition, BMI was positively correlated with E3/E2 (r=0.272, *p*=0.015), and Matsuda index was negatively correlated with E3/E2 (r=-0.317, *p*=0.004) (**Figure 2**).

## 4 Discussion

**Figure 3:**
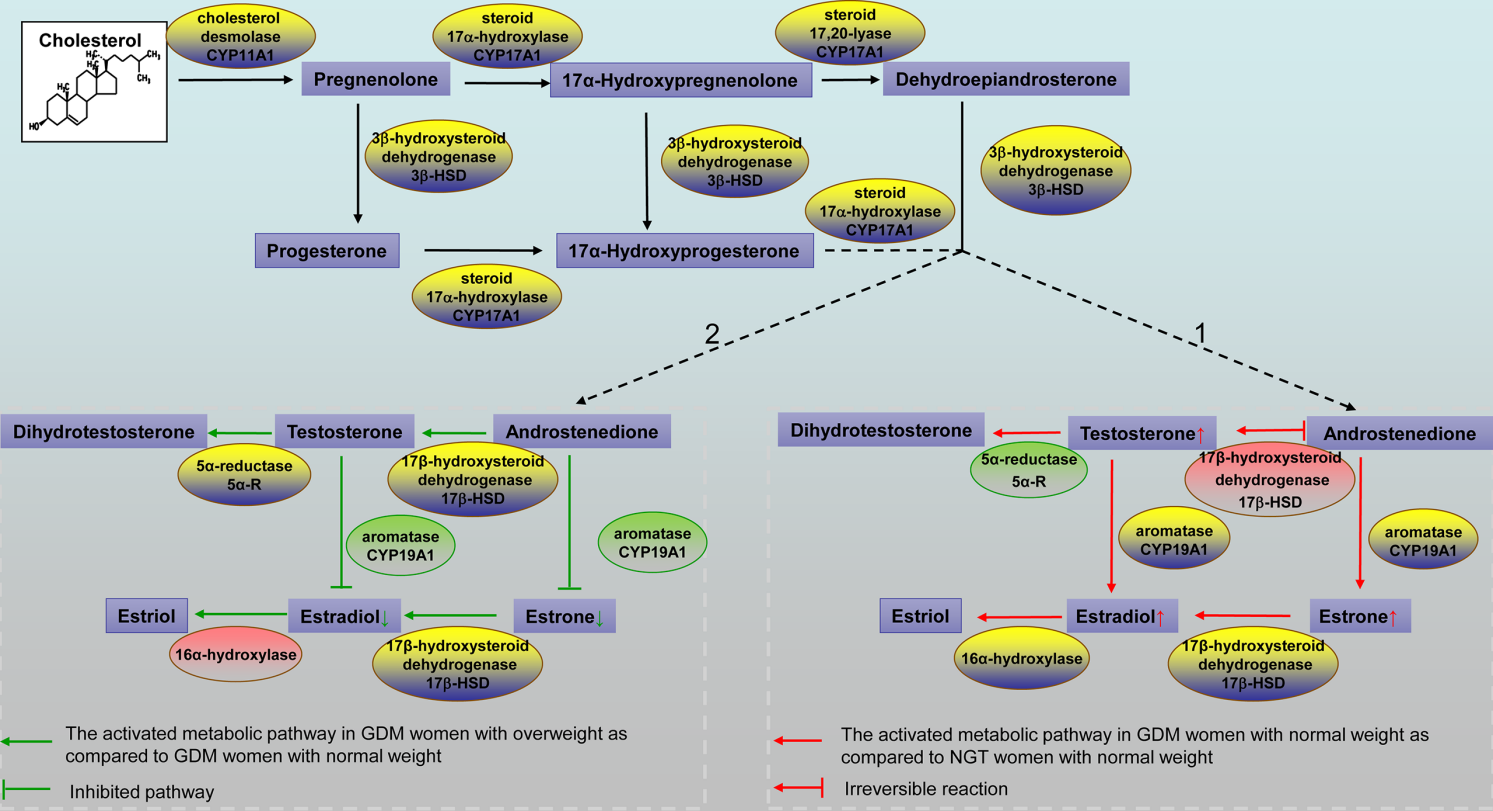
Schematic representation of the steroidogenic pathways. Pathway 1. The activated metabolic pathway in GDM women with normal weight compared to NGT women with normal weight is indicated by red arrows. Steroid hormones that showed a higher concentration in GDM women with normal weight are shown in the box with small red arrows. The red box indicates a higher product/precursor ratio in GDM women with normal weight. The green box indicates a lower product/precursor ratio in GDM women with normal weight. Pathway 2. The activated metabolic pathway in GDM women with overweight compared to GDM women with normal weight is indicated by green arrows. Steroid hormones that showed a lower concentration in women with GDM with overweight are shown in the box with small green arrows. The red box indicates a higher product/precursor ratio in GDM women with overweight. The green box indicates a lower product/precursor ratio in GDM women with overweight. GDM, gestational diabetes mellitus; NGT, normal glucose tolerance.

In this study, elevated serum glucose, insulin, HbA1c, and TG levels were expected in GDM patients, which is consistent with previous studies (27). However, there was no significant difference in maternal BMI between GDM patients and healthy pregnant women. One explanation might be that pregnant women pay greater attention to weight management, particularly to a healthy diet and maintenance/increase in physical activity (28). Steroid hormone metabolism was distinctly profiled in GDM women and NGT women, and it has been validated that BMI correlates with steroid hormone metabolism (29). We hypothesized that there would be differences based on BMI groups between women with GDM and women with NGT. We performed a comprehensive measurement of 11 known steroid hormones in the steroidogenic pathway between women with GDM and NGT using LC-MS/MS. We observed a decreased insulin sensitivity and hyperandrogenism in women with GDM compared with women with NGT. In pregnant women who were normal weight, we found a substantial alteration in androgen and estrogen synthesis between women with GDM and women with NGT. T/A4 representing 17β‐hydroxysteroid dehydrogenase (17βHSD) activity increased significantly in women with GDM than in women with NGT (Pathway 1 in **Figure 3**). Interestingly, our results also demonstrated that the differential profile of steroid hormone is correlated with BMI in women with GDM. Specifically, in GDM women with overweight, the concentrations of E1, E2, E1/A4 and E2/T decreased significantly, representing decreased activity of cytochrome P450 aromatase (CYP19A1) in the steroidogenic pathway (Pathway 2 in **Figure 3**). Thus, our results shed new light on the occurrence of GDM from the perspective of steroid hormone metabolism in pregnant women with different BMI.

Disorders of steroid hormone metabolism have been associated with insulin metabolism. A cohort studies has revealed increased serum T and DHEA levels in pregnant women with PCOS, who were always diagnosed with insulin resistance and higher BMI. Furthermore, a higher level of androgen has also been observed in GDM patients with insulin resistance (30, 31). Uzelac et al. found that women with GDM have higher serum androgen and lower estrogen levels than women without GDM in the third trimester of pregnancy. Their study suggests that owing to decreased conversion of T to estrogen and increased leptin production, the placenta of GDM patients has elevated levels of T and leptin. The underlying mechanism is that the androgen and leptin signaling pathways may be overactivated by the presence of excessive ligands and overexpressed receptors in the GDM placenta. Disorders of these two endocrine networks may lead to placental abnormalities and maternal and fetal complications associated with GDM (32). Our study confirmed elevated serum DHEA, T, and A4 levels in women with GDM. However, a latest study reveals that serum T and E2 levels is reduced with the increasing of gestational age, while DHEA, A4 and E1 were found to be unrelated to GDM (33). This finding goes against our work. One probable reason is that our study is a one timepoint study. We can only note that steroid profile differed in women with GDM and women with NGT at 24–28 gestation weeks. Another possible reason is that the abovementioned studies had a small sample size and a larger, longitudinal cohort study is needed to validate their findings.

In pregnant women who were normal weight, women with GDM showed higher DHEA, T, E1, E2, and 17β-HSD activity than women with NGT. According to recent literature, T, E3, P5, and DHEA might be the differential metabolites for GDM. The genetic variants rs10046 of CYP19A1 and rs2257157 of 17βHSD isoform 3 could predispose to GDM in Chinese women (34). Additionally, we observed a reduced Matsuda index and HOMA-β in women with GDM, which was used to evaluate insulin sensitivity and the function of pancreatic β-cells. Studies in women with PCOS have reported that androgen excess predisposes to pancreatic β-cell dysfunction, indicating inadequate insulin release or an exaggerated insulin response to glucose. In addition, β-cell dysfunction was positively correlated with T concentration, independent of insulin resistance (35, 36). In mice, knockout of androgen receptors protects them from hyperinsulinemia and insulin resistance when exposed to chronic androgen excess (37). It is possible that androgen excess is associated with pancreatic β-cell dysfunction. Therefore, we speculated that excessive androgen synthesis may impair pancreatic β-cell function and reduce insulin sensitivity, resulting in hyperglycemia in GDM women with normal weight.

During pregnancy, the biochemical synthesis of steroid hormone including estrogens, 16α-hydroxylation, and aromatization requires interacting processing in the placenta, the fetal and maternal adrenal glands, and the fetal liver. This interdependent physiological entity is known as the feto-placental unit, which is involved in steroid hormone synthesis and metabolism (38). Within this unit, the fetal adrenal gland can synthesize steroid hormone precursors–DHEAS that can be used by the placenta to produce estrogens. DHEAS can be converted into 16α­hydroxy­DHEAS (16OH­DHEAS) and 15,16OH­DHEAS by 15α­hydroxylase and 16α­ hydroxylase in the fetal liver. Maternal DHEAS is further catabolized by the placenta to E1 and E2, whilst the placenta converts 16OH­DHEAS to E3, respectively (39). Fetal adrenal hypertrophy and DHEA production is promoted by adrenocorticotropic hormone (ACTH), which is secreted by the fetal pituitary gland. A previous study showed that pregnant women with an anencephalic fetus (in which levels of ACTH secreted from the fetal pituitary gland are markedly reduced), the levels of circulating E3 are very low as a result of impaired development of the fetal zone (40). Fetal adrenal hypoplasia is a rare condition that presents as marked low maternal serum levels of E3 during the second trimester (41). In our study, the last antenatal care recorded is normal for all subjects. In combination with stringent requirements for inclusion, differences in estrogen levels due to abnormal placental unit were excluded.

In the early second trimester of pregnancy, high concentrations of unconjugated E3 in the maternal serum have been considered to be a useful predictor of GDM development (42). However, our results revealed that different steroid hormone metabolism exist between GDM women with overweight and normal weight. GDM women with overweight showed a reduced in E1 and E2 levels, increased insulin levels, and decreased insulin sensitivity. Aromatase activity is related to estrogen generation in the placenta during pregnancy. We found that lower CYP19A1 activities was related to higher BMI and declined insulin sensitivity in GDM women with overweight. A previous finding indicates that aromatase availability in the amygdala is negatively associated with BMI. It also demonstrated that individual variations in the brain’s capacity for estrogen synthesis may influence the risk of obesity and self-control (43). Previous findings have revealed a novel role for E2 in the regulation of energy metabolism and glucose homeostasis. Aromatase knockout mice have decreased E2 levels, accompanied by reduced glucose oxidation, elevated adiposity, and insulin levels (44, 45). E2 is an important antidiabetic steroid operating *via* binding to nuclear receptors as well as *via* modulation of ion channels controlling the secretion of pancreatic hormones (33, 46). Therefore, E2 deficiency in GDM women with overweight may be an important component participating in the pathophysiology of GDM.

In conclusion, NGT women and GDM women with normal weight presented with different steroid hormone profiles. Steroidogenic pathway profiling of sex hormone synthesis showed a significant increase in the production of DHEA, T, E1, and E2 in GDM women with normal weight. Additionally, the alteration of steroid hormone metabolism was related to maternal BMI in women with GDM, and GDM women with overweight showed reduced estrogen production and declined insulin sensitivity compared with GDM women with normal weight.

Our novel finding suggests that steroid hormone metabolic changes need to be considered in GDM development, especially in GDM patients with different BMI. We believe that our study makes a significant contribution to the GDM research. However, our study is limited by the small sample population and missed clinical outcomes, and a larger longitudinal cohort research is needed in the future to validate our results. In addition, more cellular protein mechanistic studies are needed for further study.

## Data availability statement

The raw data supporting the conclusions of this article will be made available by the authors, without undue reservation.

## Ethics statement

The studies involving human participants were reviewed and approved by Ethics Committee of Women’s Hospital School of Medicine, Zhejiang University. Written informed consent for participation was not required for this study in accordance with the national legislation and the institutional requirements. Written informed consent was obtained from the individual(s) for the publication of any potentially identifiable images or data included in this article.

## Author contributions

YS, BZ designed the study and drafted the manuscript. XM, BY collected the data. KW, YL performed statistical data analyses. DZo, JX performed steroid hormones measurement. XS, DZh and ZM contributed to revising the manuscript. All authors contributed to the article and approved the submitted version.

## Funding

This work was supported by the National Key Research and Development Program of China (2018YFC1005003); National Natural Science Foundation of China (No. 81974224, 81771535); Key Research and Development Program of Zhejiang Province (2021C03098); Science and Technology Program of Zhejiang Province (2022C01061) and Zhejiang University Education Foundation Global Partnership Fund.

## Acknowledgments

We thank all the physicians, technicians, especially the patients involved in their dedication to the study.

## Conflict of interest

Authors DZ and JX were employed by Hangzhou BIOZON Medical Laboratory Co.

The remaining authors declare that the research was conducted in the absence of any commercial or financial relationships that could be construed as a potential conflict of interest.

## Publisher’s note

All claims expressed in this article are solely those of the authors and do not necessarily represent those of their affiliated organizations, or those of the publisher, the editors and the reviewers. Any product that may be evaluated in this article, or claim that may be made by its manufacturer, is not guaranteed or endorsed by the publisher.

## Abbreviations

GDM, Gestational diabetes; T2DM, type 2 diabetes mellitus; BMI,body mass index; PCOS, polycystic ovary syndrome; DHEAS, dehydroepiandrosterone sulfate; NGT, normal glucose tolerant; LC-MS/MS, Liquid chromatography-tandem mass spectrometry; HOMA, Homeostasis model assessment; TG, triacylglycerol; P5, Pregnenolone; P4, Progesterone; 17OHP5, 17α-hydroxypregnenolone; 17OHP4, 17α-hydroxyprogesterone; DHEA, Dehydroepiandrosteron; A4, Androstenedion; T, Testosterone; DHT, Dihydrotestosterone; E3, Estriol; E2, Estradiol; E1, Estrone; 17βHSD, 17β‐hydroxysteroid dehydrogenase; CYP19A1, cytochrome P450 aromatase.
