# Supplementary material for: Effect of maternal body mass index on the steroid profile in women with gestational diabetes mellitus
Source: Front Endocrinol (Lausanne). 2022 Nov 9;13:999154. doi: 10.3389/fendo.2022.999154 (PMC9681895; doi:10.3389/fendo.2022.999154)

**Effect of maternal body mass index on the steroid profile in women with gestational diabetes mellitus**

**Supplementary Materials**

Yanni Sun,^1,2#^ Bo Zhu,^1,2#^ Xingjun Meng,^1,2^Binbin Yin,^1,2^ Kaiqi Wu,^1,2^ Yifeng Liu,^1,3^ Dandan Zou,^4^ Jianyou Xue,^4^ Xiao Sun^1,3^, Dan Zhang^*1,3^ Zhixin Ma^*1,2,^

^1^Women’s Hospital, School of Medicine, Zhejiang University, Hang Zhou, China.

^2^Clinical Prenatal Diagnosis Center, Women’s Hospital, School of Medicine, Zhejiang University, China.

^3^Key Laboratory of Women's Reproductive Health of Zhejiang Province, and Women's Hospital, School of Medicine, Zhejiang University, Hangzhou, China.

^4^Hangzhou BIOZON Medical Laboratory co. LTD, Hangzhou, Zhejiang, China

^#^These authors contribute equally to the work.

^*^Corresponding author

Corresponding author: Zhixin Ma (11418122@zju.edu.cn) and Dan Zhang (zhangdan@zju.edu.cn)

Postal address: Women’s Hospital, School of Medicine, Zhejiang University, Xueshi Road 2#, Hang Zhou, 310006, China.

Table1. The concentration(ng/mL) of steroid hormones

| steroid hormones | calibrator 1 | calibrator 2 | calibrator 3 | calibrator 4 | calibrator 5 | calibrator 6 | calibrator 7 | calibrator 8 |
| --- | --- | --- | --- | --- | --- | --- | --- | --- |
| Dihydrotestosterone | 5 | 1 | 0.5 | 0.1 | 0.05 | 0.01 | 0.005 | 0.0025 |
| Estrone | 5 | 1 | 0.5 | 0.1 | 0.05 | 0.01 | 0.005 | 0.0025 |
| Estradiol | 20 | 4 | 2 | 0.4 | 0.2 | 0.04 | 0.02 | 0.01 |
| Androstenedione | 20 | 4 | 2 | 0.4 | 0.2 | 0.04 | 0.02 | 0.01 |
| Pregnenolone | 20 | 4 | 2 | 0.4 | 0.2 | 0.04 | 0.02 | 0.01 |
| Dehydroepiandrosterone | 50 | 10 | 5 | 1 | 0.5 | 0.1 | 0.05 | 0.025 |
| Testosterone | 50 | 10 | 5 | 1 | 0.5 | 0.1 | 0.05 | 0.025 |
| Estriol | 50 | 10 | 5 | 1 | 0.5 | 0.1 | 0.05 | 0.025 |
| 17α-hydroxyprogesterone | 50 | 10 | 5 | 1 | 0.5 | 0.1 | 0.05 | 0.025 |
| 17α-hydroxypregnenolone | 150 | 30 | 15 | 3 | 1.5 | 0.3 | 0.15 | 0.075 |
| Progesterone | 200 | 40 | 20 | 4 | 2 | 0.4 | 0.2 | 0.1 |

Table2. The concentration(ng/mL) of steroid hormones internal standard

| internal standard | concentration (ng/mL) |
| --- | --- |
| Dihydrotestosterone -d3 | 1 |
| Estrone-d4 | 1 |
| Estradiol-d3 | 4 |
| Androstenedione-C3 | 4 |
| Pregnenolone-d4 | 4 |
| Dehydroepiandrosterone -d5 | 10 |
| Testosterone-d3 | 10 |
| Estriol-d3 | 10 |
| 17α-hydroxyprogesterone -C2H2 | 10 |
| 17α-hydroxypregnenolone -d8 | 30 |
| Progesterone-d9 | 40 |

Table 3. The concentration(ng/mL) of steroid hormones QC

| steroid hormones | QCL | QCM | QCH |
| --- | --- | --- | --- |
| Dihydrotestosterone | 0.025 | 0.3 | 3.75 |
| Estrone | 0.025 | 0.3 | 3.75 |
| Estradiol | 0.1 | 1.2 | 15 |
| Androstenedione | 0.1 | 1.2 | 15 |
| Pregnenolone | 0.1 | 1.2 | 15 |
| Dehydroepiandrosterone | 0.25 | 3 | 37.5 |
| Testosterone | 0.25 | 3 | 37.5 |
| Estriol | 0.25 | 3 | 37.5 |
| 17α-hydroxyprogesterone | 0.25 | 3 | 37.5 |
| 17α-hydroxypregnenolone | 0.75 | 9 | 112.5 |
| Progesterone | 0.1 | 12 | 150 |

Supplementary Table 4. The details of recovery experiment.

| Steroid hormone | Sample | Group A | Group B | Recovery |
| --- | --- | --- | --- | --- |
| Androstenedione | L1 | 0.095 | 0.095 | 99.79% |
|  | L2 | 0.092 | 0.097 | 94.73% |
|  | L3 | 0.096 | 0.096 | 99.79% |
|  | M1 | 3.036 | 3.149 | 96.42% |
|  | M2 | 3.027 | 3.171 | 95.47% |
|  | M3 | 3.018 | 3.162 | 95.46% |
|  | H1 | 4.224 | 4.134 | 102.17% |
|  | H2 | 4.200 | 4.145 | 101.31% |
|  | H3 | 4.206 | 4.151 | 101.30% |
| Testosterone | L1 | 0.243 | 0.241 | 100.79% |
|  | L2 | 0.236 | 0.246 | 95.97% |
|  | L3 | 0.249 | 0.248 | 100.24% |
|  | M1 | 8.021 | 7.978 | 100.54% |
|  | M2 | 8.014 | 8.008 | 100.08% |
|  | M3 | 8.140 | 7.963 | 102.22% |
|  | H1 | 11.071 | 10.463 | 105.81% |
|  | H2 | 11.084 | 10.467 | 105.89% |
|  | H3 | 11.058 | 10.430 | 106.01% |
| Dehydroepiandrosterone | L1 | 0.207 | 0.211 | 98.25% |
|  | L2 | 0.217 | 0.212 | 102.69% |
|  | L3 | 0.208 | 0.213 | 97.70% |
|  | M1 | 1.035 | 1.060 | 97.65% |
|  | M2 | 0.991 | 0.979 | 101.25% |
|  | M3 | 1.036 | 1.085 | 95.52% |
|  | H1 | 1.559 | 1.578 | 98.80% |
|  | H2 | 1.608 | 1.518 | 105.98% |
|  | H3 | 1.638 | 1.542 | 106.21% |
| Progesterone | L1 | 0.332 | 0.346 | 95.90% |
|  | L2 | 0.375 | 0.355 | 105.64% |
|  | L3 | 0.355 | 0.355 | 99.86% |
|  | M1 | 12.443 | 12.095 | 102.87% |
|  | M2 | 12.664 | 12.194 | 103.85% |
|  | M3 | 12.549 | 12.207 | 102.80% |
|  | H1 | 17.673 | 15.993 | 110.51% |
|  | H2 | 17.526 | 15.937 | 109.97% |
|  | H3 | 17.528 | 15.417 | 113.69% |
| Pregnenolone | L1 | 0.463 | 0.444 | 104.33% |
|  | L2 | 0.449 | 0.473 | 95.01% |
|  | L3 | 0.499 | 0.470 | 106.30% |
|  | M1 | 1.374 | 1.370 | 100.31% |
|  | M2 | 1.363 | 1.348 | 101.13% |
|  | M3 | 1.408 | 1.320 | 106.69% |
|  | H1 | 2.480 | 2.523 | 98.28% |
|  | H2 | 2.509 | 2.569 | 97.67% |
|  | H3 | 2.527 | 2.524 | 100.11% |
| 17α-hydroxyprogesterone | L1 | 0.004 | 0.004 | 87.80% |
|  | L2 | 0.003 | 0.004 | 85.00% |
|  | L3 | 0.004 | 0.004 | 92.31% |
|  | M1 | 0.109 | 0.116 | 93.30% |
|  | M2 | 0.110 | 0.117 | 94.11% |
|  | M3 | 0.110 | 0.119 | 92.02% |
|  | H1 | 0.155 | 0.157 | 98.60% |
|  | H2 | 0.155 | 0.156 | 99.23% |
|  | H3 | 0.157 | 0.154 | 102.08% |
| 17α-hydroxypregnenolone | L1 | 0.439 | 0.520 | 84.45% |
|  | L2 | 0.475 | 0.500 | 94.82% |
|  | L3 | 0.492 | 0.505 | 97.44% |
|  | M1 | 21.860 | 20.297 | 107.70% |
|  | M2 | 22.451 | 20.604 | 108.96% |
|  | M3 | 21.814 | 20.450 | 106.67% |
|  | H1 | 28.108 | 28.407 | 98.95% |
|  | H2 | 30.204 | 28.069 | 107.61% |
|  | H3 | 30.714 | 28.632 | 107.27% |
| Dihydrotestosterone | L1 | 0.248 | 0.247 | 100.57% |
|  | L2 | 0.245 | 0.258 | 94.99% |
|  | L3 | 0.262 | 0.248 | 105.74% |
|  | M1 | 1.897 | 1.787 | 106.13% |
|  | M2 | 1.967 | 1.759 | 111.82% |
|  | M3 | 2.091 | 1.802 | 116.04% |
|  | H1 | 9.999 | 9.230 | 108.34% |
|  | H2 | 9.434 | 9.920 | 95.10% |
|  | H3 | 9.599 | 9.956 | 96.41% |
| Estrone | L1 | 0.055 | 0.052 | 104.60% |
|  | L2 | 0.053 | 0.051 | 103.95% |
|  | L3 | 0.050 | 0.050 | 99.12% |
|  | M1 | 0.215 | 0.214 | 100.66% |
|  | M2 | 0.224 | 0.210 | 106.56% |
|  | M3 | 0.225 | 0.208 | 107.92% |
|  | H1 | 0.323 | 0.284 | 113.59% |
|  | H2 | 0.314 | 0.288 | 108.85% |
|  | H3 | 0.306 | 0.319 | 96.20% |
| Estradiol | L1 | 0.055 | 0.057 | 95.78% |
|  | L2 | 0.052 | 0.057 | 91.42% |
|  | L3 | 0.053 | 0.053 | 99.64% |
|  | M1 | 1.654 | 1.741 | 95.02% |
|  | M2 | 1.616 | 1.715 | 94.26% |
|  | M3 | 1.726 | 1.682 | 102.63% |
|  | H1 | 2.426 | 2.398 | 101.15% |
|  | H2 | 2.282 | 2.333 | 97.81% |
|  | H3 | 2.579 | 2.554 | 100.96% |
| Estriol | L1 | 0.068 | 0.065 | 104.48% |
|  | L2 | 0.069 | 0.071 | 97.03% |
|  | L3 | 0.067 | 0.062 | 107.68% |
|  | M1 | 0.214 | 0.193 | 110.77% |
|  | M2 | 0.206 | 0.203 | 101.58% |
|  | M3 | 0.204 | 0.192 | 106.42% |
|  | H1 | 0.305 | 0.298 | 102.48% |
|  | H2 | 0.275 | 0.301 | 91.07% |
|  | H3 | 0.287 | 0.317 | 90.41% |

Extraction recoveries were evaluated on the basis of two groups. For group A, three different concentrations (low, mid and high) of each steroid hormones were spiked into steroid-free serum (Fitzgerald Industries International, Inc. USA) before extraction, and the internal standard mixture was added to the organic extract after extraction. For group B, both steroid hormones and internal standard mixture were added after extraction. Extraction recoveries were calculated by dividing the peak areas ratios of steroids to the internal standard in group A to the ratios in group B. The measured values were considered acceptable when they were between 85% and 115%.

Table 5. The details of matrix effect.

| Steroid hormone | Sample | Serum | Low | | | Mid | | | High | | |
| --- | --- | --- | --- | --- | --- | --- | --- | --- | --- | --- | --- |
|  |  |  | 10% methanol | 1:1 mix | Matrix effect | 10% methanol | 1:1 mix | Matrix effect | 10% methanol | 1:1 mix | Matrix effect |
| Androstenedione | 1 | 4.734 | 0.227 | 2.721 | 9.70% | 3.080 | 3.732 | -4.47% | 33.292 | 19.406 | 2.07% |
|  | 2 | 1.385 |  | 0.820 | 1.77% |  | 1.893 | -15.22% |  | 18.237 | 5.18% |
|  | 3 | 3.376 |  | 1.766 | -1.99% |  | 2.927 | -9.31% |  | 18.646 | 1.70% |
|  | 4 | 3.486 |  | 2.013 | 8.43% |  | 2.989 | -8.95% |  | 18.925 | 2.91% |
|  | 5 | 5.102 |  | 2.610 | -2.05% |  | 3.790 | -7.37% |  | 21.338 | 11.15% |
|  | 6 | 4.043 |  | 2.203 | 3.21% |  | 3.150 | -11.54% |  | 18.846 | 0.95% |
| Testosterone | 1 | 0.830 | 1.307 | 1.097 | 2.62% | 14.035 | 7.497 | 0.86% | 151.148 | 87.915 | 15.69% |
|  | 2 | 0.439 |  | 0.886 | 1.46% |  | 7.472 | 3.25% |  | 90.922 | 19.96% |
|  | 3 | 1.152 |  | 1.237 | 0.61% |  | 7.828 | 3.09% |  | 83.875 | 10.14% |
|  | 4 | 19.060 |  | 10.293 | 1.08% |  | 16.778 | 1.39% |  | 92.512 | 8.70% |
|  | 5 | 2.147 |  | 1.739 | 0.67% |  | 9.434 | 16.60% |  | 85.852 | 12.01% |
|  | 6 | 24.975 |  | 13.388 | 1.88% |  | 18.720 | -4.03% |  | 99.565 | 13.06% |
| Dehydroepiandrosterone | 1 | 0.421 | 0.052 | 0.263 | 11.30% | 0.344 | 0.387 | 1.07% | 4.911 | 2.610 | -2.10% |
|  | 2 | 0.068 |  | 0.065 | 8.81% |  | 0.211 | 2.38% |  | 2.500 | 0.45% |
|  | 3 | 0.123 |  | 0.092 | 4.90% |  | 0.235 | 0.70% |  | 2.717 | 7.96% |
|  | 4 | 0.241 |  | 0.162 | 10.50% |  | 0.280 | -4.27% |  | 2.773 | 7.65% |
|  | 5 | 0.391 |  | 0.255 | 15.16% |  | 0.364 | -1.04% |  | 2.811 | 6.02% |
|  | 6 | 0.196 |  | 0.147 | 18.25% |  | 0.321 | 18.70% |  | 2.540 | -0.55% |
| Progesterone | 1 | 0.00030 | 0.00320 | 0.00166 | -5.48% | 0.03258 | 0.01706 | 3.78% | 0.33949 | 0.19075 | 12.28% |
|  | 2 | 0.00006 |  | 0.00154 | -5.50% |  | 0.01696 | 3.95% |  | 0.19853 | 16.94% |
|  | 3 | 0.00007 |  | 0.00151 | -7.77% |  | 0.01709 | 4.70% |  | 0.20169 | 18.79% |
|  | 4 | 0.00009 |  | 0.00152 | -7.58% |  | 0.01685 | 3.19% |  | 0.19501 | 14.85% |
|  | 5 | 0.00008 |  | 0.00155 | -5.70% |  | 0.01533 | -6.10% |  | 0.19044 | 12.17% |
|  | 6 | 0.00009 |  | 0.00157 | -4.75% |  | 0.01591 | -2.58% |  | 0.19105 | 12.52% |
| Pregnenolone | 1 | 0.059 | 0.006 | 0.036 | 10.72% | 0.047 | 0.048 | -9.47% | 0.733 | 0.357 | -9.78% |
|  | 2 | 0.002 |  | 0.005 | 15.19% |  | 0.025 | 2.52% |  | 0.362 | -1.45% |
|  | 3 | 0.008 |  | 0.006 | -14.13% |  | 0.027 | -2.22% |  | 0.319 | -13.95% |
|  | 4 | 0.014 |  | 0.009 | -11.30% |  | 0.027 | -9.43% |  | 0.352 | -5.62% |
|  | 5 | 0.012 |  | 0.009 | 1.15% |  | 0.032 | 7.77% |  | 0.384 | 3.18% |
|  | 6 | 0.014 |  | 0.009 | -8.59% |  | 0.024 | -21.42% |  | 0.410 | 9.90% |
| 17α-hydroxyprogesterone | 1 | 0.011 | 0.002 | 0.007 | 10.48% | 0.025 | 0.018 | 2.53% | 0.375 | 0.194 | 0.39% |
|  | 2 | 0.002 |  | 0.002 | 4.64% |  | 0.014 | 3.74% |  | 0.196 | 3.94% |
|  | 3 | 0.002 |  | 0.002 | 8.25% |  | 0.014 | -0.87% |  | 0.197 | 4.62% |
|  | 4 | 0.010 |  | 0.006 | 3.84% |  | 0.018 | 0.32% |  | 0.197 | 1.96% |
|  | 5 | 0.004 |  | 0.004 | 19.04% |  | 0.014 | -5.71% |  | 0.184 | -3.02% |
|  | 6 | 0.013 |  | 0.008 | 1.55% |  | 0.018 | -6.73% |  | 0.194 | -0.31% |
| 17α-hydroxypregnenolone | 1 | 0.018 | 0.002 | 0.012 | 14.70% | 0.024 | 0.020 | -5.05% | 0.371 | 0.204 | 4.94% |
|  | 2 | 0.000 |  | 0.001 | -9.72% |  | 0.013 | 9.99% |  | 0.186 | -0.02% |
|  | 3 | 0.001 |  | 0.002 | 9.99% |  | 0.013 | 5.88% |  | 0.192 | 3.19% |
|  | 4 | 0.008 |  | 0.005 | 5.98% |  | 0.016 | 0.08% |  | 0.189 | -0.21% |
|  | 5 | 0.010 |  | 0.006 | -4.05% |  | 0.015 | -11.51% |  | 0.176 | -7.51% |
|  | 6 | 0.005 |  | 0.004 | 18.77% |  | 0.016 | 15.39% |  | 0.182 | -3.19% |
| Dihydrotestosterone | 1 | 0.168 | 0.584 | 0.384 | 2.23% | 6.359 | 3.304 | 1.25% | 73.280 | 41.772 | 13.75% |
|  | 2 | 0.056 |  | 0.317 | -0.78% |  | 3.249 | 1.29% |  | 43.703 | 19.19% |
|  | 3 | 0.091 |  | 0.346 | 2.49% |  | 3.264 | 1.20% |  | 43.566 | 18.76% |
|  | 4 | 0.439 |  | 0.514 | 0.46% |  | 3.540 | 4.14% |  | 40.976 | 11.17% |
|  | 5 | 0.170 |  | 0.386 | 2.48% |  | 3.625 | 11.04% |  | 42.546 | 15.85% |
|  | 6 | 1.905 |  | 1.246 | 0.10% |  | 4.014 | -2.85% |  | 43.566 | 15.89% |
| Estrone | 1 | 0.014 | 0.040 | 0.027 | 0.37% | 0.479 | 0.220 | -10.75% | 6.351 | 3.156 | -0.83% |
|  | 2 | 0.005 |  | 0.024 | 6.67% |  | 0.276 | 14.05% |  | 3.440 | 8.24% |
|  | 3 | 0.005 |  | 0.020 | -11.11% |  | 0.231 | -4.55% |  | 3.362 | 5.79% |
|  | 4 | 0.003 |  | 0.026 | 19.07% |  | 0.217 | -9.96% |  | 3.336 | 5.00% |
|  | 5 | 0.010 |  | 0.026 | 4.00% |  | 0.216 | -11.66% |  | 3.205 | 0.77% |
|  | 6 | 0.010 |  | 0.024 | -4.00% |  | 0.255 | 4.29% |  | 3.418 | 7.47% |
| Estradiol | 1 | 0.001 | 0.004 | 0.003 | 8.00% | 0.071 | 0.040 | 11.11% | 1.211 | 0.548 | -9.57% |
|  | 2 | 0.002 |  | 0.003 | -16.67% |  | 0.040 | 9.59% |  | 0.566 | -6.68% |
|  | 3 | 0.001 |  | 0.003 | 0.00% |  | 0.038 | 5.56% |  | 0.567 | -6.44% |
|  | 4 | 0.001 |  | 0.002 | -16.00% |  | 0.037 | 2.78% |  | 0.540 | -10.89% |
|  | 5 | 0.001 |  | 0.002 | -4.00% |  | 0.039 | 8.33% |  | 0.643 | 6.11% |
|  | 6 | 0.001 |  | 0.003 | 4.00% |  | 0.039 | 8.33% |  | 0.570 | -5.94% |
| Estriol | 1 | 0.007 | 0.006 | 0.008 | 18.46% | 0.067 | 0.043 | 16.22% | 0.917 | 0.479 | 3.68% |
|  | 2 | 0.011 |  | 0.009 | 5.88% |  | 0.046 | 17.95% |  | 0.452 | -2.59% |
|  | 3 | 0.005 |  | 0.006 | 9.09% |  | 0.041 | 13.89% |  | 0.485 | 5.21% |
|  | 4 | 0.004 |  | 0.006 | 12.00% |  | 0.039 | 9.86% |  | 0.483 | 4.89% |
|  | 5 | 0.002 |  | 0.004 | 0.00% |  | 0.034 | -1.45% |  | 0.464 | 0.98% |
|  | 6 | 0.001 |  | 0.003 | -14.29% |  | 0.037 | 8.82% |  | 0.444 | -3.27% |

Matrix effect: Low, mid, and high concentrations of steroid hormone were dissolved in 10% methanol and 90% water and selected for injection analysis without extraction. Six biological matrix samples were selected for injection analysis after extraction. For the measurement of the 1:1 mixed solution sample, three different concentrations of steroid hormone (dissolved in 10% methanol and 90% water) were 1:1 mixed with extracted biological matrix sample for injection analysis. If the difference between the response value of the 1:1 mixed solution sample and the mean value of the response value of the biological matrix sample and three different concentrations of steroid hormone is less than a certain proportion (20%), it proves that the presence of matrix effect does not affect the accurate quantification of the target analyte.

Table 6. Multiple reaction monitoring (MRM) of 11 steroid hormones quantification list.

MRM of 11 steroid hormones

| **No.** | **Compound Name** | **molecular ion (m/z)** | **fragment ion (m/z)** | **Cone voltage (kv)** | **Collision energy (eV)** | **Ionization mode** |
| --- | --- | --- | --- | --- | --- | --- |
| **Method A** | | | | | | |
| 1 | Dehydroepiandrosterone | 271.16 | 213.09 | 45 | 12 | ES+ |
|  | Dehydroepiandrosterone -d5 | 276.2 | 218.06 | 46 | 12 | ES+ |
| 2 | Androstenedione | 287.26 | 97.08 | 24 | 30 | ES+ |
|  | Androstenedione-C3 | 290.26 | 100.1 | 24 | 20 | ES+ |
| 3 | Testosterone | 289.33 | 109.09 | 40 | 15 | ES+ |
|  | Testosterone-d3 | 292.2 | 109.1 | 40 | 20 | ES+ |
| 4 | 17α-hydroxyprogesterone | 315.2 | 297.1 | 30 | 25 | ES+ |
|  | 17α-hydroxyprogesterone-C2H2 | 319.2 | 301.1 | 30 | 10 | ES+ |
| 5 | 17α -hydroxypregnenolone | 331.25 | 109.5 | 40 | 40 | ES+ |
|  | 17α -hydroxypregnenolone -d8 | 339.42 | 100.1 | 40 | 32 | ES+ |
| 6 | Dihydrotestosterone | 291.4 | 255.4 | 45 | 16 | ES+ |
|  | Dihydrotestosterone -d3 | 294.2 | 258.5 | 45 | 17 | ES+ |
| 7 | Pregnenolone | 299.2 | 159.1 | 32 | 20 | ES+ |
|  | Pregnenolone-d4 | 303.2 | 163.1 | 32 | 20 | ES+ |
| 8 | Progesterone | 315.2 | 109.1 | 45 | 70 | ES+ |
|  | Progesterone-d9 | 324.2 | 100.1 | 40 | 40 | ES+ |
| **Method B** | | | | | | |
| 9 | Estrone | 269.1 | 145.3 | 40 | 38 | ES- |
|  | Estrone-d4 | 273.1 | 147.3 | 40 | 38 | ES- |
| 10 | Estradiol | 271.3 | 145.3 | 40 | 38 | ES- |
|  | Estradiol-d3 | 274.3 | 185.1 | 40 | 38 | ES- |
| 11 | Estriol | 287 | 143.03 | 40 | 38 | ES- |
|  | Estriol-d3 | 290 | 173.07 | 40 | 38 | ES- |

Figure 1. The ratio of E3/E2, E/A4 and E2/T in NGT women (BMI<25kg/m^2^) and NGT women (BMI>25kg/m^2^). Androstenedione (A4), Testosterone (T), Estriol (E3), Estradiol (E2), Estrone (E1).


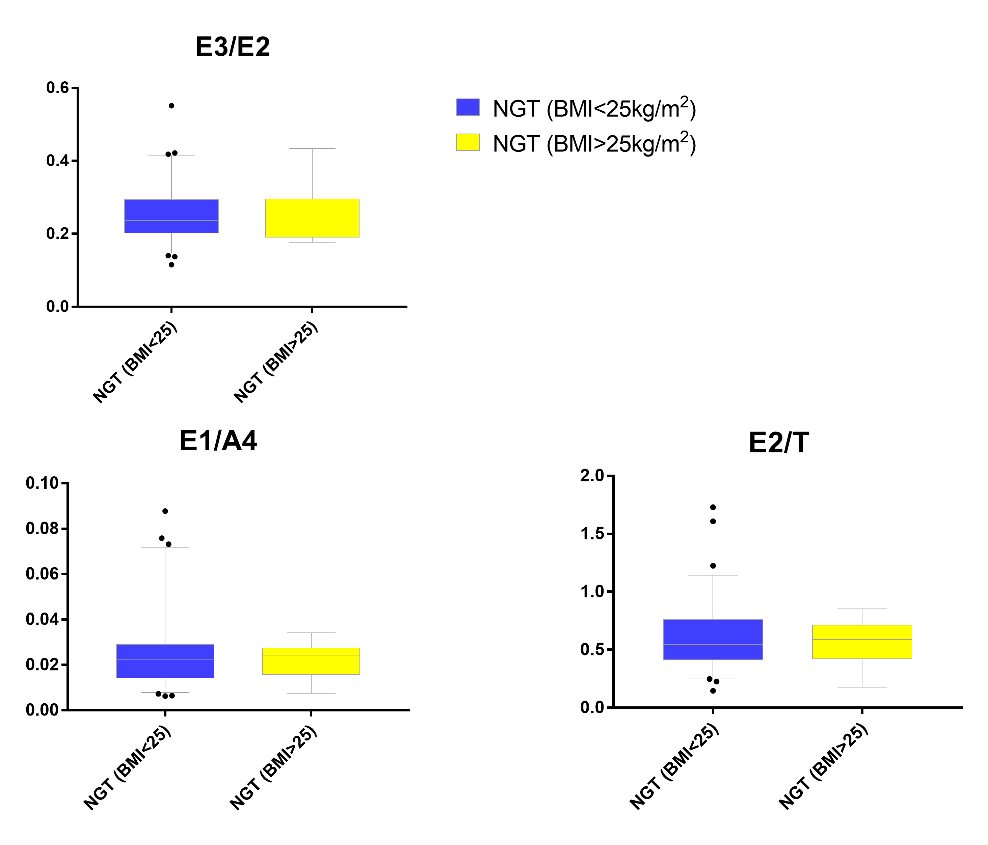

Supplement: Supplementary file 1 [file DataSheet_1.docx]
